# Relationship between Poorly Controlled Asthma and Sleep-Related Breathing Disorders in Children with Asthma: A Two-Center Study

**DOI:** 10.1155/2021/8850382

**Published:** 2021-01-28

**Authors:** Yun Guo, Xiuqing Zhang, Feng Liu, Ling Li, Deyu Zhao, Jun Qian

**Affiliations:** ^1^Department of Respiratory Medicine, The Affiliated Wuxi Children's Hospital of Nanjing Medical University, Wuxi, China; ^2^Department of Respiratory Medicine, Children's Hospital of Nanjing Medical University, Nanjing, China; ^3^Department of Respiratory Medicine, Tianjin Children's Hospital, Tianjin, China

## Abstract

**Objective:**

Asthma and sleep-related breathing disorders (SRBD) are common chronic respiratory diseases in children. The relationship between asthma and SRDB is bidirectional. However, only a few studies have analyzed the relationship between asthma control status and risk of SRBD. The aim of this study was to evaluate the relationship between asthma control and SRBD and further assess the relationship between therapy/atopy/lung function of children with asthma and SRBD.

**Methods:**

A total of 209 children aged 3–16 years were enrolled in this study. Pediatric sleep questionnaire (PSQ) scores were used to identify children at high risk of developing SRBD. Data on asthma control status, therapy, allergy, lung function, and exhaled nitric oxide were collected.

**Results:**

A significantly higher risk of SRBD was found among children with poorly controlled asthma (34.25% vs. 13.97%, *P* < 0.01) and allergic rhinitis (AR) (34.29% vs. 13.92%, *P* < 0.01) than among children with well-controlled asthma and AR. The prevalence of SRBD was also significantly higher in asthmatic children with obesity than that with just obesity (42.11% vs. 20.00%, *P* < 0.05). Multiple logistic regression analysis showed that poorly controlled asthma (OR, 2.746 (95% CI, 1.215–6.209); *P* < 0.05) and poorly controlled AR (OR, 3.284 (95% CI, 1.430–7.544); *P* < 0.01) increased the odds of having SRBD.

**Conclusion:**

Poorly controlled asthma and AR increase the risk of SRBD. A routine check of the level of asthma control and appropriate use of medication for AR are important because of their influence on SRBD.

## 1. Introduction

Asthma is a serious global health problem that affects 2.4–37.6% of the population across various countries [1]. Over the past few decades, the prevalence of asthma has rapidly increased in several countries, including China [2]. In China, the first national cross-sectional study, conducted in 1990, showed that the average prevalence of asthma was 0.91% [3]; however, this value increased to 1.54% in 2000 and 3.02% in 2010 [4, 5]. Asthma control is commonly associated with exacerbations, reduced quality of life, poor school attendance, and sleep-related breathing disorders (SRBD).

SRBD are common in childhood; its spectrum ranges from primary snoring to partial or complete airway obstruction, which is termed obstructive sleep apnea syndrome (OSAS) [6]. SRBD affects 5.1–13.3% of children [7–16], resulting in dysfunction of immune responses, cardiovascular function, and neurocognitive function [6]. Recently, research has shown that asthma is associated with a high risk of SRBD in children. A systematic review of 17 studies that included 45,155 children showed that asthmatic children have 1.91 times higher odds of reporting symptoms that suggest SRBD [9]. Another meta-analysis of 12 studies that included 38,766 subjects showed that SRBD is correlated with the prevalence of asthma in children (OR, 1.58; 95% CI, 1.35–1.80) [14]. Zandieh et al. enrolled 9,565 adolescents in their study and found that adolescents with asthma have 2.63 times higher odds of developing SRBD than those without asthma [6].

Asthma may impair sleep via several mechanisms. It can alter circadian patterns of gene expression, which play an important role in SRBD processes and severity. Asthma is also associated with airway inflammation and resistance, which cause decreased airway flow rates during sleep, thereby affecting the quality of sleep [17, 18]. Furthermore, common comorbidity of asthma such as obesity, gastroesophageal reflux, and allergic rhinitis (AR) may also lead to SRDB due to narrowing of the upper airway by local fat infiltration [15], altered parasympathetic tone to trigger bronchoconstriction [13], and nasal congestion [16]. These mechanisms provide evidence that the prevalence of asthma is associated with a high risk of SRBD in children. However, only few studies focusing on the pediatric population have reported on the relationship between asthma control/management and SRBD. Perikleous et al. [16] demonstrated that poor asthma control was associated with SRBD, and asthma control test scores were negatively correlated with pediatric sleep questionnaire (PSQ) scores; however, a limitation of this study was that only 9 children were poorly controlled, which was a small number. In addition, no data are available about the relationship between asthma control and SRDB in China to date.

The present study aimed to evaluate the relationship between asthma control and SRBD in China and further assess the relationship between therapy/atopy/lung function of children with asthma and SRBD.

## 2. Materials and Methods

### 2.1. Ethics Approval and Consent to Participate

This study was conducted in accordance with the amended Declaration of Helsinki. Ethical approval was provided by the medical ethics committee of Wuxi Children's Hospital (No. WXCH2019-8-002). Informed consent was obtained from all individual participants included in the study and for participants under 16 years of age, and written informed consent was obtained from a parent.

### 2.2. Participants

A total of 209 children aged 3–16 years who visited the Asthma Outpatient Unit of Nanjing Children's Hospital, Jiangsu, China, and Wuxi Children's Hospital, Jiangsu, China, for routine follow-up from June 1, 2019 to June 30, 2019 were enrolled in this study. Both hospitals are tertiary specialist hospitals for children. Since a strict appointment and referral system has not been implemented for children's medical treatment in China, in these two hospitals, appointments can be made directly in the asthma clinic without referral. Asthma outpatient service consults with the deputy chief physician or chief physician of the department of respiratory medicine. The diagnosis and management of asthma was in accordance with the guidelines of the Global Initiative for Asthma (GINA) [2]. The guidelines for the diagnosis and treatment of allergic rhinitis and the clinical practice guidelines for the diagnosis and treatment of AR in pediatric patients were used to diagnose AR and evaluate AR control status [19, 20]. A total score of 5 or greater of the visual analog scale for the combined nasoocular symptoms was defined as uncontrolled AR [21, 22].

### 2.3. Asthma Control Status

When children with asthma visited the asthma outpatient unit, an allergy specialist determined whether the patient had “well-controlled,” “partly controlled,” or “uncontrolled” asthma according to the GINA guidelines [2]. GINA assessment of asthma control consists of 4 questions regarding asthma symptoms and therapy in the past 4 weeks. Daytime asthma symptoms more than twice/week? Any night waking due to asthma? Short-acting *β*2-agonist reliever for symptoms more than twice/week? Any activity limitation due to asthma? If the answer was “yes” to none of the questions, their asthma was defined as well-controlled. If the answer was “yes” to 1-2 of the questions, it was defined as partly controlled, while if the answer was “yes” to 3-4 of the questions, it was defined as uncontrolled. Children with “partly controlled” and “uncontrolled” asthma were then classified into a “poorly controlled” asthma group.

### 2.4. Sociodemography

General sociodemographic data (sex, age, weight (kg), and height (cm)) were collected. Weight and height were measured by a nurse. Body mass index (BMI) was calculated as weight (kg)/height^2^ (m^2^). The diagnosis of obesity was according to BMI reference norm for screening overweight and obesity in Chinese [23]. The survey included information on the child's therapeutic regimen such as the use of inhaled corticosteroid, montelukast, antihistamines, allergen immunotherapy (AIT), and nasal steroids. Allergy information such as past diagnosis of AR, AR control status, and allergy tests were collected from their medical chart. In addition, data on lung function and exhaled nitric oxide (FeNO) (if tested) were also collected from the medical chart.

### 2.5. Sleep Data

The PSQ was used to assess the scale of SRBD. The PSQ identifies children with SRBD with a sensitivity of 83% and a specificity of 87%; its applicability and generalizability in assessing childhood SRBD in China has been confirmed [24]. This multipage questionnaire consists of 22 questions on snoring, sleepiness, and behavioral problems and has been used frequently for sleep-associated respiratory research studies [15, 16, 24–31]. The three possible responses to each question are “yes = 1,” “no = 0,” and “do not know = missing.” The number of symptom items endorsed positively (“yes”) is divided by the number of items answered positively or negatively; the denominator therefore excludes items with missing responses, i.e., items answered with “do not know.” Scores >0.33 are considered positive and suggestive of a high risk of having SRBD. The questionnaire requires parents to assess the sleep acts of their children during the past month. Children were excluded from the study if more than 30% of the items in the PSQ were missing.

### 2.6. Statistical Analysis

*R* × 64 3.6.0 and RStudio for Windows (Auckland, New Zealand) were used for data processing. Normally distributed data were expressed as mean ± standard deviation. Nonnormally distributed data were represented as the median and interquartile range (IQR). The *t*-test was used to analyze the differences between groups if the group variances were homogeneous, whereas the Mann–Whitney *U* test was used to analyze the differences between groups if the group variances were heterogeneous. Pearson's chi-squared test or Fisher's exact test was used to analyze categorical variables. Stepwise logistic regression was performed to reduce the questionnaire items to the set of items that were risk or protective factors for SRBD. A *P* value of 0.1 was selected a priori as the criterion for item retention, and finally, six variables were included in the analyses. Odds ratios (ORs) and 95% confidence intervals (CIs) were calculated by logistic regression.

## 3. Results

During the study, 247 children with asthma visited the Asthma Outpatient Unit of Nanjing Children's Hospital and Wuxi Children's Hospital for routine follow-up; 209 children met the inclusion criteria and were enrolled in this study. Out of the enrolled children, 149 (71.29%) patients had AR. Out of the 38 excluded children, 8 had chronic conditions other than atopic diseases, 17 were excluded because they were younger than 3 years old or older than 16 years old, 6 were excluded because of missing values in the PSQ, and 7 were excluded because their parents were unwilling to participate in the study.

According to categorization based on the GINA guidelines [2], 136 (65.07%) children had well-controlled asthma. The demographic, comorbidity, and lung function characteristics of the study population categorized based on asthma control status are shown in Table 1. Out of the demographic characteristics, age (7.61 (IQR, 5.71–10.11) vs. 6.27 (IQR, 4.87–8.54); *P* < 0.05) was associated with a well-controlled asthma status. BMI, obesity, the history of AR, atopy, and AIT did not significantly differ between the patients with well-controlled and poorly controlled asthma. Children in the well-controlled asthma group had lower PSQ sores (0.13 (IQR 0.04–0.26) vs. 0.22 (IQR 0.13–0.35); *P* < 0.001) and with a SRBD risk (13.97% vs. 34.24%; *P* < 0.01) than children in the poorly controlled asthma group.

Out of the 209 included patients, 44 (21.05%) had SRBD; the characteristics, comorbidities, medications, and lung functions of the enrolled children categorized based on presence of SRBD are listed in Table 2. A significantly higher risk of SRBD was found among children with poorly controlled asthma (34.25% vs. 13.97%, *P* < 0.01) and poorly controlled AR (34.29% vs. 13.92%, *P* < 0.01) than among those with well-controlled asthma and well-controlled AR. The prevalence of obesity was also significant higher in patients with SRDB than those without SRDB (42.11% vs. 20.00%, *P* < 0.05). However, other demographic characteristics, therapy characteristics, lung functions, and FeNO did not significantly differ between patients with and without SRBD.

Logistic regression analysis was performed to evaluate the risk factors for SRBD in children with asthma. Univariate logistic regression analysis showed that the incidence of SRBD increased in the case of obesity (OR, 3.111 (95% CI, 1.176–8.292); *P* < 0.05), poorly controlled asthma (OR, 3.207 (95% CI, 1.617–6.360); *P* < 0.01), and poorly controlled AR (OR, 3.225 (95% CI, 1.441–7.220); *P* < 0.01). Age, the history of AR, atopy, and therapy characteristics did not affect the odds of having SRBD. Obesity, asthma, and AR control status were finally included in the multiple logistic regression analysis (*P* < 0.05). Poorly controlled asthma (OR, 2.746 (95% CI, 1.215–6.209); *P* < 0.05) and poorly controlled AR (OR, 3.284 (95% CI, 1.430–7.544); *P* < 0.01) increased the odds of having SRBD (Table 3).

## 4. Discussion

The main finding of this study was that the risk of SRBD was significantly higher in children with poorly controlled asthma (34.25% vs. 13.97%) and poorly controlled AR (34.29% vs. 13.92%) than in children with well-controlled asthma and AR. Poorly controlled asthma (OR, 2.851 (95% CI, 1.269–6.406), *P* < 0.05) and poorly controlled AR (OR, 3.363 (95% CI, 1.470–7.693); *P* < 0.01) increased the odds of having SRBD.

The overall rate of well-controlled asthma in the present study was 65.07%; this rate is better than that in the 2015-2016 asthma control data for China (28.65%) [32]. This could be due to the establishment of the China Asthma Alliance, which improved the treatment of the disease as well as the management of comorbidities and education of patients and their families [33, 34]. However, the history of AR and atopy did not significantly differ between the patients with well-controlled and poorly controlled asthma, considering that it is related to the higher proportion of allergic children using antiallergy treatment including antihistamines and AIT (data not shown).

Obesity is an independent factor for both asthma and SRDB, and the relationship between obesity and SRDB is well known [10, 35]. In addition, obesity is a stronger link between SRDB and asthma. Zidan et al. [36] found a higher BMI was found to be a significant independent predictor of the development of OSA in asthmatics. We also evaluated the prevalence of obesity in our children and found a higher SRDB prevalence in children with obesity than in those without obesity. In the present study, we found a 3.1-fold increased risk for SRBD among asthmatic children with obesity; however, after adjusting with asthma control status and AR control status, the logistic regression analysis of the effect of obesity on SRDB yielded negative results. Obesity may impair sleep via several mechanisms. It can modify the anatomy and collapsibility of the upper airways by local fat infiltration and a greater effort in breathing due to increase in body fat [15]. Obesity can also influence ventilation control and increase respiratory workload [37]. Furthermore, a variety of inflammatory cytokines and adipocyte-specific cytokines such as adiponectin and leptin in serum of obese children are elevated, making the children in a state of systemic inflammation, which plays an important role in the processes and severity of SRDB [38, 39].

The relationship between asthma and SRBD has garnered some attention in recent years. In the present study, we found that the risk of SRBD in children with asthma was 21.05%. This is in line with the results of previous pediatric studies [1, 10–12, 15, 16, 27] but higher than the results of epidemiological surveys, which reported prevalence rates from 5.1% to 13.3% [7–16]. We also found that partly controlled or uncontrolled asthma was a risk factor for SRBD in children; coexistence of SRBD (diagnosed based on PSQ scores) was significantly more frequent in children with poorly controlled asthma than in those with well-controlled asthma (30.09% vs. 15.67%). Our findings are concordant with those of previous studies, which show a positive association between SRBD and asthma control. Perikleous et al. [16] evaluated 140 children (65 with asthma, 57 with both asthma and AR, and 18 with only AR), using PSQ and found a negative association between the results of childhood asthma control test scores and PSQ scores. In another study of 408 children with asthma aged 4–18 years, children with poorly controlled asthma had a higher prevalence of SRBD than children with well-controlled asthma (59.6% vs. 18.2%) [28]. However, only a few studies have assessed the correlation between the risk for SRBD and asthma control. In the present study, we found a 3.2-fold increased risk for SRBD among children with poorly controlled asthma. Furthermore, poorly controlled asthma increased the risk for SRDB independently of AR and obesity after adjusted with AR and obesity; poorly controlled asthma also increased the odds of having SRBD (OR, 2.746 (95% CI, 1.215–6.209); *P* < 0.05).

It is worth noting that PSQ is more helpful in children with nocturnal symptoms to understand the sleep status of children with well-controlled asthma who still having nocturnal symptoms; we also assessed the sleep patterns of enrolled children categorized according to asthma control status and found that children with uncontrolled asthma were more likely to have the following responses in the PSQ: “have ‘heavy' or loud breathing,” “have trouble breathing or struggle to breathe,” “breathe through the mouth during the day,” “wake up feeling unrefreshed in the morning,” “have a problem with sleepiness during the day,” “hard to wake your child up in the morning,” “easily distracted by extraneous stimuli,” “fidgets with hands or feet or squirms in seat,” and “interrupts or intrudes on others” (Supplementary Table 1). These results suggest that children with poorly controlled asthma have a significantly higher proportion of nocturnal symptoms related to sleep disorders than children with well-controlled asthma and further support that poorly controlled asthma-related airway inflammation and resistance may cause decreased airway flow rates during sleep, thereby affecting the quality of sleep [17, 18]. In addition, a previous study found that children with poorly controlled asthma had later bedtimes, had longer sleep onset latency, and had more frequent and longer nocturnal awakenings than children with well-controlled asthma [40]; our study data are in line with and supplements these observations.

It is known that asthma and AR usually coexist. In the present study, we found that 71.29% of the comorbidities reported were AR; the prevalence of AR was not different between the children with and without SRBD. However, we found that uncontrolled AR and SRBD in children with asthma were strongly related. A 3.2-fold increased risk of having SRBD was found among children with poorly controlled AR, a finding which is consistent with those of previous studies [41, 42]. Nasal obstruction, especially caused by AR, has long been considered as one of the leading risk factors for upper airway obstruction during sleep [42]. However, rhinitis responsible for oral breathing and nocturnal symptoms, which in the children, needs to be distinguished from nocturnal events of SRDB. We assessed the sleep patterns of the enrolled children categorized according to AR control status (Supplementary Table 2). These results showed that children with uncontrolled AR were more likely to have the following responses in the PSQ: “Snore more than half the time,” “Snore loudly,” “Stop breathing during the night,” “Breathe through the mouth during the day,” “Have a dry mouth on waking the in morning,” and “wake up feeling unrefreshed in the morning.” The mechanism of AR-related SRDS was complex. Allergic inflammation of the nasal mucosa will result in nasal congestion, which may lead to oral breathing patterns and sleep fragmentation and may also lead to an increased risk of asthma exacerbation. Chronic rhinosinusitis and adenoid hypertrophy are common accompanying diseases in children with AR, which may be one of the reasons why AR is a high risk factor for sleep disordered breathing [30, 43–45]. Hence, nasal congestion is a treatable cause of SRBD in children [13]. Treatment with intranasal budesonide for six weeks effectively reduces the severity of mild OSAS [46]. We also evaluated the usage of nasal steroids and found that only 4.03% of children with AR use nasal steroids regularly; we did not find a significant difference between the children with and without SRBD. The small sample size may be responsible for the absence of a relationship between nasal steroids and the prevalence SRBD.

Recent studies have demonstrated that allergic sensitization is associated with decreased pulmonary function and increased asthma morbidity [47, 48]. We explored the relationship between allergic sensitization and SRBD in the present study and found a slightly higher prevalence of allergy among children with SRBD than among those without SRBD; however, there was no significant difference between the two groups. Esteban et al. [49] found that children who were sensitized to aeroallergens, cockroaches, cats, dust mites, or dogs had lower overall sleep efficiency, more variability in their sleep efficiency, and more night awakenings. A limitation of this study is that we did not analyze the details of allergens, and this may have led to our overall negative association.

As antihistamines are always required for children with allergies, we assessed the relationship between antihistamines and SRBD, but no correlation was found between the two. This may be because all the enrolled children were taking new-generation antihistamines, which have little effect on the central nervous system. AIT is a therapy for asthma or AR patients who are sensitized to dust mites; it can significantly modify the disease in a clinically relevant manner [41]. We also assessed the possible role of AIT treatment in SRBD and found that the percentage of children who underwent AIT treatment was slightly higher in the SRBD group than in the non-SRBD group (13.64% vs. 7.88%); however, there was no significant difference between the two groups. This may be because children who undergo AIT always develop severe and persistent AR; moreover, this may be associated with a potential recruitment bias.

According to the GINA guidelines, lung function is important for determining the level of asthma control; however, the relationship between lung function and SRBD is unclear. In a previous study, Sheen et al. [25] enrolled 220 children with asthma and revealed that FEV1/forced vital capacity is associated with the PSQ score. However, another research showed that there is no difference between the lung functions of patients with a high risk of SRBD and those of patients with a low risk of SRBD [16]. In the present study, we did not find a relationship between lung function and SBD. A plausible reason for this is that many children with uncontrolled asthma have normal lung function, and lung function does not correlate strongly with asthma symptoms in children [2, 50].

This study has some limitations that should be noted. Since children with asthma who were classified as low risk of developing SRBD according to PSQ were found to have SRBD via polysomnography [29], this study may be associated with a potential risk of biased results as SRBD diagnosis was based on PSQ results. In addition, asthma control status was assessed according to GINA guidelines; however, questionnaires such as the childhood asthma control test and asthma control questionnaire were not used. Regarding allergic sensitization, we did not analyze the details of allergens. Another missing aspect in this work is the consideration of gastroesophageal reflux, which is well known as a risk factor for both diseases in children, and gastroesophageal reflux was also associated with SRDS because of trigger bronchoconstriction through altered parasympathetic tone or other mechanisms [13]. Such information may help to elucidate other predictive factors for SRBD.

## 5. Conclusion

The risk of SRBD is significantly higher in children with poorly controlled asthma and AR. Children with uncontrolled asthma are more likely to have poor sleep quality and sleep-associated problems than children with well-controlled asthma. Poorly controlled asthma and poorly controlled AR increase the odds of having SRBD. We suggest that clinicians should routinely inquire about the level of asthma control for each child with asthma. Appropriate use of medication to control AR is also important because of its influence on SRBD.

## Figures and Tables

**Table 1:** General characteristics of enrolled children according to asthma control status.

Characteristics	Well-controlled asthma (*n* = 136)	Partly or uncontrolled asthma (*n* = 73)	Group differences	*P* values
**Age, median (min-max)**	**7.61 (5.71–10.11)**	**6.27 (4.87–8.54)**	***Z*** **=** −**2.294**	**0.022**

Male, *n* (%)	82 (60.29)	39 (53.42)	*χ* ^2^ = 0.920	0.338

BMI, median (min-max)	16.28 (14.77–18.31)	16.42 (14.88–19.79)	*Z* = −0.358	0.719

Obesity, *n* (%)	9 (6.62)	10 (13.70)	*χ* ^2^ = 1.423	0.233

History of AR, *n* (%)	99 (72.79)	50 (68.49)	*χ* ^2^ = 0.429	0.512

Allergy, *n* (%)	122 (89.71)	68 (93.15)	*χ* ^2^ = 0.682	0.409

**PSQ, median (min-max)**	**0.13 (0.04–0.26)**	**0.22 (0.13–0.35)**	***Z*** **=** −**4.180**	**≤0.001**

**SRBD, *n* (%)**	**19 (13.97)**	**25 (34.24)**	***χ*** ^**2**^ **=** **11.750**	**0.001**

Lung function				
FVC (% pred) median (min-max)	85.30 (79.55–94.50)	84.10 (74.40–97.90)	*Z* = -1.564	0.118
**FEV1 (% pred) median (min-max)**	**97.00 (93.35–107.95)**	**91.90 (85.40–104.20)**	***Z*** **=** **-2.678**	**0.007**
FEV1/FVC median (min-max)	113.90 (110.90–116.10)	113.40 (107.40–116.10)	*Z* = −1.294	0.196
**PEF (% pred) median (min-max)**	**97.00 (84.15–108.50)**	**83.90 (76.60–95.00)**	***Z*** **=** −**3.930**	**≤0.001**

FeNO median (min-max)	14.00 (10.00–23.50)	13.00 (10.00–18.00)	*Z* = −0.159	0.874

Data were presented as mean ± SD, median, min-max, or *n* (%) unless otherwise stated. *P* < 0.05 was considered a statistically significant difference. BMI, body mass index; AR, allergic rhinitis; PSQ, pediatric sleep questionnaire; SRBD, sleep-related breathing disorders; FeNO, exhaled nitric oxide; SD, standard deviation; FEV, forced vital capacity; FEV1, forced expiratory volume in 1 second; PEF, peak expiratory flow; min-max, minimum and maximum interquartile range. The variables with statistical differences are indicated in bold.

**Table 2:** Characteristics, comorbidities, and medications of enrolled children according to SRBD.

Characteristics	SRBD (*n* = 44)	Non-SRBD (*n* = 165)	Group differences	*P* values
Age, median (min-max)	6.90 (5.11–9.27)	7.15 (5.37–9.59)	*Z* = -0.255	0.798

Male, *n* (%)	27 (61.36)	94 (56.97)	*χ* ^2^ = 0.275	0.600

BMI, median (min-max)	16.68 (15.15–21.77)	16.26 (14.71–18.23)	*Z* = -1.316	0.188

**Obesity (*n*** **=** **19), *n* (%)**	**8 (42.11)**	**11 (20.00)**	***χ*** ^**2**^ **=** **5.573**	**0.018**

**Asthma, *n* (%)**				
**Well-controlled**	**19 (13.97)**	**117 (86.03)**		
**Partly or uncontrolled**	**25 (34.25)**	**48 (65.75)**	***χ*** ^**2**^ **=** **11.750**	**0.001**

History of AR, *n* (%)	35 (23.49)	114 (76.51)	*χ* ^2^ = 1.855	0.173
**Well-controlled**	**11 (13.92)**	**68 (86.08)**		
**Partly or uncontrolled**	**24 (34.29)**	**46 (65.71)**	***χ*** ^**2**^ **=** **8.562**	**0.003**

Allergy, *n* (%)	42 (22.11)	148 (77.89)	*χ* ^2^ = 1.393	0.238

Therapy				
ICS, *n* (%)	39 (88.64)	143 (86.67)	*χ*2 = 0.120	0.729
Nasal steroids, *n* (%)	2 (4.55)	4 (2.42)	*χ*2 = 0.154	0.695
Montelukast, *n* (%)	37 (84.09)	129 (78.18)	*χ*2 = 0.742	0.389
Antihistamines, *n* (%)	29 (65.91)	105 (63.64)	*χ*2 = 0.078	0.860
AIT *n* (%), (*n* = 223)	6 (13.64)	13 (7.88)	*χ* ^2^ = 1.403	0.236

Lung function				
FVC (% pred) median (min-max)	85.35 (76.20–98.80)	85.10 (78.90–93.75)	*Z* = −0.163	0.871
FEV1 (% pred) median (min-max)	92.85 (86.70–111.40)	96.70 (88.65–107.05)	*Z* = −0.877	0.381
FEV1/FVC median (min-max)	112.05 (106.40–115.20)	113.90 (110.70–116.20)	*Z* = −1.176	0.239
PEF (% pred) median (min-max)	88.40 (77.30–100.00)	93.90 (82.50–104.35)	*Z* = −0.858	0.391

FeNO median (min-max)	13.00 (9.00–18.00)	14.00 (10.00–22.00)	*Z* = −0.716	0.474

Data were presented as mean ± SD, median, min-max, or *n* (%) unless otherwise stated. *P* < 0.05 was considered a statistically significant difference. SRDB, sleep-related breathing disorders; BMI, body mass index; AR, allergic rhinitis; FeNO, exhaled nitric oxide; ICS, inhaled corticosteroid; AIT, allergen immunotherapy; SD, standard deviation; FEV, forced vital capacity; FEV1, forced expiratory volume in 1 second; PEF, peak expiratory flow; min-max, minimum and maximum interquartile range. The variables with statistical differences are indicated in bold.

**Table 3:** Risk factors for SRBD in asthmatic children.

Characteristic	Univariate analysis		Multivariate analysis	
	OR (95% CI)	*P* values	OR (95% CI)	*P* values
Age in years	1.000 (0.905–1.105)	1.000		

**Obesity**				
**No**	**1**	**0.023**	**1.995 (0.658–6.050)**	**0.223**
**Yes**	**3.111 (1.176–8.292)**			

**Asthma**				
**Well-controlled**	**1**		**1**	
**Partly or uncontrolled**	**3.207 (1.617–6.360)**	**0.001**	**2.746 (1.215–6.209)**	**0.015**

History of AR				
No	1			
Yes	1.740 (0.779–3885)	0.177		

**AR control status**		**0.004**		**0.005**
**Controlled**	**1**		**1**	
**Uncontrolled**	**3.225 (1.441–7.220)**		**3.284 (1.430–7.544)**	

Allergy		0.251		
No	1			
Yes	2.412 (0.536–10.861)			

ICS therapy		0.730		
No	1			
Yes	1.200 (0.427–3.373)			

Nasal steroids therapy		0.696		
No	1			
Yes	1.417 (0.247–8.133)			

Montelukast therapy		0.391		
No	1			
Yes	1.475 (0.607–3.586)			

Antihistamines therapy		0.780		
No	1			
Yes	1.105 (0.549–2.223)			

The data from multivariate analysis were after adjusting for obesity, asthma control status, and AR control status. *P* < 0.05 was considered a statistically significant difference. CI, confidence interval; OR, odds ratios; AR, allergic rhinitis; ICS, inhaled corticosteroid. The variables with statistical differences are indicated in bold.

## Data Availability

The datasets generated and/or analyzed during the current study are not publicly available because data do not have consent from all patients to share their information online but are available from the corresponding author upon request.

## Supplementary Materials

Supplementary Materials Supplementary Table 1. Sleep patterns of enrolled children according to asthma control status. Supplementary Table 2. Sleep patterns of enrolled children according to AR control status.
