# Supplementary material for: Relationship between Poorly Controlled Asthma and Sleep-Related Breathing Disorders in Children with Asthma: A Two-Center Study
Source: Can Respir J. 2021 Jan 28;2021:8850382. doi: 10.1155/2021/8850382 (PMC7861934; doi:10.1155/2021/8850382)
Supplement: Supplementary Materials — Supplementary Table 1. Sleep patterns of enrolled children according to asthma control status. Supplementary Table 2. Sleep patterns of enrolled children according to AR control status. [file 8850382.f1.docx]

Supplementary table 1 Sleep patterns of enrolled children according to asthma control status

| characteristics | Well-controlled asthma (n=136) | Partly- or un-controlled asthma（n=73） | Group differences | P Values |
| --- | --- | --- | --- | --- |
| Snore more than half the time | 19 (13.97) | 15 (20.55) | χ^2^=1.509 | 0.219 |
| Always snore | 11 (8.09) | 9 (12.33) | χ^2^=0.987 | 0.320 |
| Snore loudly | 12 (8.82) | 10 (13.70) | χ^2^=1.199 | 0.274 |
| **Have ``heavy'' or loud breathing** | **35 (25.74)** | **29 (39.73)** | **χ^2^=4.377** | **0.036** |
| **Have trouble breathing, or struggle to breathe** | **10 (7.35)** | **13 (17.80)** | **χ^2^=5.302** | **0.035** |
| Stop breathing during the night | 3 (2.21) | 2 (2.74) | χ^2^=0.058 | 0.810 |
| **Breathe through the mouth during the day** | **17 (12.50)** | **21 (28.77)** | **χ^2^=8.450** | **0.004** |
| Have a dry mouth on waking the in morning | 47 (34.56) | 34 (46.58) | χ^2^=2.890 | 0.089 |
| Occasionally wet the bed | 24 (17.65) | 12 (16.44) | χ^2^=0.049 | 0.825 |
| **Wake up feeling unrefreshed in the morning** | **36 (26.47)** | **35 (47.95)** | **χ^2^=9.766** | **0.002** |
| **Have a problem with sleepiness during the day** | **12 (8.82)** | **14 (19.18)** | **χ^2^=4.676** | **0.031** |
| Appears sleepy during the day | 10 (7.35) | 7 (9.59) | χ^2^=0.318 | 0.573 |
| **Hard to wake your child up in the morning** | **43 (31.62)** | **36 (49.32)** | **χ^2^=6.328** | **0.012** |
| Wake up with headaches in the morning | 5 (3.68) | 0 (0.00) | χ^2^=2.750 | 0.097 |
| Stop growing at a normal rate | 29 (21.32) | 17 (23.29) | χ^2^=0.107 | 0.730 |
| Overweight | 8 (6.25) | 10 (13.70) | χ^2^=3.687 | 0.055 |
| Not seem to listen when spoken to directly | 37 (27.21) | 25 (34.24) | χ^2^=1.129 | 0.288 |
| Has difficulty organizing task and activities | 7 (5.15) | 6 (8.22) | χ^2^=0.769 | 0.383 |
| **Easily distracted by extraneous stimuli** | **32 (23.53)** | **34 (46.58)** | **χ^2^=11.677** | **0.001** |
| **Fidgets with hands or feet or squirms in seat** | **44 (32.35)** | **39 (53.42)** | **χ^2^=8.810** | **0.003** |
| Often acts as if “driven by a motor” | 46 (33.82) | 31 (42.47) | χ^2^=1.526 | 0.217 |
| **Interrupts or intrudes on others** | **46 (33.82)** | **35 (47.95)** | **χ^2^=3.991** | **0.046** |

Data were presented as n(%) unless otherwise stated. P <0.05 was considered statistically significant difference.

Supplementary table 2 Sleep patterns of enrolled children according to AR control status

| characteristics | Controlled AR (n=79) | Un-controlled AR（n=70） | Group differences | P Values |
| --- | --- | --- | --- | --- |
| **Snore more than half the time** | **8 (10.13)** | **16 (22.86)** | **χ^2^=4.451** | **0.035** |
| Always snore | 5 (6.76) | 10 (14.29) | χ^2^=2.595 | 0.107 |
| **Snore loudly** | **2 (2.53)** | **13 (18.57)** | **χ^2^=10.547** | **0.002** |
| Have ``heavy'' or loud breathing | 22 (27.85) | 27 (38.57) | χ^2^=1.934 | 0.164 |
| Have trouble breathing, or struggle to breathe | 7 (8.86) | 13 (18.57) | χ^2^=3.012 | 0.083 |
| **Stop breathing during the night** | **0 (0.00)** | **5 (7.14)** | **χ^2^=5.800** | **0.021** |
| **Breathe through the mouth during the day** | **12 (15.19)** | **20 (28.57)** | **χ^2^=3.941** | **0.047** |
| **Have a dry mouth on waking the in morning** | **23 (29.11)** | **36 (51.43)** | **χ^2^=7.727** | **0.005** |
| Occasionally wet the bed | 11 (13.92) | 13 (18.57) | χ^2^=0.593 | 0.441 |
| **Wake up feeling unrefreshed in the morning** | **23 (29.11)** | **32 (45.71)** | **χ^2^=4.392** | **0.036** |
| Have a problem with sleepiness during the day | 7 (8.86) | 11 (15.71) | χ^2^=1.641 | 0.218 |
| Appears sleepy during the day | 5 (6.33) | 10 (14.28) | χ^2^=2.595 | 0.107 |
| Hard to wake your child up in the morning | 25 (31.65) | 29 (41.43) | χ^2^=1.537 | 0.215 |
| Wake up with headaches in the morning | 1 (1.27) | 4 (5.71) | χ^2^=2.249 | 0.187 |
| Stop growing at a normal rate | 16 (20.25) | 13 (18.57) | χ^2^=0.067 | 0.796 |
| Overweight | 7 (8.86) | 8 (11.43) | χ^2^=0.270 | 0.603 |
| Not seem to listen when spoken to directly | 21 (26.58) | 24 (34.29) | χ^2^=1.045 | 0.307 |
| Has difficulty organizing task and activities | 5 (6.33) | 7 (10.00) | χ^2^=0.675 | 0.411 |
| Easily distracted by extraneous stimuli | 26 (32.91) | 27 (38.57) | χ^2^=0.519 | 0.471 |
| Fidgets with hands or feet or squirms in seat | 33 (41.77) | 31 (44.29) | χ^2^=0.096 | 0.757 |
| Often acts as if “driven by a motor” | 23 (29.11) | 30 (42.86) | χ^2^=3.059 | 0.080 |
| Interrupts or intrudes on others | 28 (35.44) | 31 (44.29) | χ^2^=1.213 | 0.271 |

Data were presented as n(%) unless otherwise stated. P <0.05 was considered statistically significant difference. AR, Allergic Rhinitis.
